# Upregulating miR-637 aggravates endoplasmic reticulum stress-induced apoptosis in gastric cancer cells by suppressing Calreticulin

**DOI:** 10.1080/19768354.2020.1816579

**Published:** 2020-09-10

**Authors:** Qingli Kong, Zhisheng Zhang, Zhipeng Liang

**Affiliations:** Department of Hepatobiliary Gastrointestinal Surgery, Tianjin Fourth Central Hospital, The Fourth Central Hospital Affiliated to Nankai University, The Fourth Center Clinical College of Tianjin Medical University, Tianjin City, People’s Republic of China

**Keywords:** MiR-637, endoplasmic reticulum stress, apoptosis, gastric cancer, Calreticulin

## Abstract

Gastric cancer is a leading cause of cancer death worldwide. Endoplasmic reticulum (ER) stress-induced apoptosis has been confirmed to be important in the treatment of gastric cancer. MiR-637 has recently been found to exert inhibitory effects on gastric cancer, and this study aimed to investigate whether miR-637 could regulate apoptosis through ER stress. The results showed that tunicamycin (TM) induced downregulation of miR-637 in gastric cancer cells (AGS) and increase of apoptosis and ER stress. Overexpression of miR-637 promoted TM-induced apoptosis and expression of ER stress associated proteins (GRP78 and CHOP), but inhibited expression of Calreticulin. MiR-637 could bind with the 3ʹ-UTR of *CALR*, and negatively regulated the expression of CALR. The co-transfection of miR-637 and CALR in AGS cells show that, CALR overexpression could reverse the pro-apoptosis effects of miR-637 in TM-treated cells. In conclusion, the present study suggests that miR-637 participates in ER stress-induced apoptosis in gastric cancer cells by suppressing CALR expression. miR-637 or CALR may be a future potential target for gastric cancer treatment.

## Introduction

Gastric cancer, one of the most common malignancies, is a leading cause of cancer death worldwide. The incidence and mortality of gastric cancer in China are higher than those in developed countries and regions (Hartgrink et al. 2009). However, there is limited clinical therapy for this highly malignant tumor (Bar-Zeev et al. 2018). Chemotherapy is still the main comprehensive treatment, but with serious adverse reactions (Hohenberger and Gretschel 2003). Therefore, it is of great significance to clarify the pathogenesis of gastric cancer and search for efficient treatment strategies. Recently, endoplasmic reticulum (ER) stress has attracted researchers’ attention and ER stress-induced apoptosis has been proposed as an effective method of cancer treatment (Reed 2003; Wang et al. 2020).

ER, an organelle responsible for the synthesis and secretion of proteins, has been identified as a subcellular compartment related with apoptosis recently (Elena Gazzano et al. 2018). Many stress conditions, such as hypoxia and defects of calcium homeostasis can lead to accumulation of unfolded and/or misfolded proteins in the ER and cause ER stress and activate the unfolded protein response (UPR) (Gorbatyuk et al. 2020). If UPR is successful or ER stress is moderate, cellular homeostasis will be restored and cells survive. However, When ER stress destroys the protective cellular responses, apoptosis ensues. The sensors of ERS or UPR can further serve to initiate pro-apoptotic transcription factors leading to apoptotic cell death (Li et al. 2006). Calreticulin (CALR) is a high capacity Ca2+ binding protein of ER and sarcoplasmic reticulum (SR). CALR has been found to regulate a variety of cellular functions, such as maintaining the homeostasis of Ca2+, assisting the folding of protein, and regulating apoptosis, through regulating the ER Ca2+ storage or acting as an ER molecular chaperone (Dudek and Michalak 2018).

MicroRNAs (miRNAs), small noncoding RNAs about 18–22 bp in length, are involved in regulating various biological processes including cell proliferation, differentiation, development and apoptosis. Many miRNAs have been confirmed to be important in the development and progression of most cancers through binding with 3’-UTR region of target genes (Wang and Lee 2009). MiR-637 has recently been found to exert inhibitory effects on gastric cancer. MiR-637 can inhibit proliferation of cholangiocarcinoma, and inhibit proliferation and metastasis of tumor cells by targeting Smad3 (Zhang et al. 2018). But little is known about the effects of miR-637 on ERS and apoptosis of cancer cells. Our study aimed to investigate whether miR-637 could regulate apoptosis of gastric cancer cells through ERS, and what is the target gene of miR-637 in regulating ERS-induced apoptosis.

## Materials and methods

### Cell culture

Human gastric cancer cells (AGS), purchased from American Type Culture collection (Rockville, MD, USA) (CRL-1739), were cultured in F-12 K medium supplemented with fetal bovine serum (FBS, 10%) and Blasticidin (with final concentration of 8 µg/mL).

### Cell viability assay

Cell viability was analyzed by Cell Counting Kit-8 (NO.C0037, Beyotime, ShangHai, China) according to the manufacturer’s instructions. Cells were seeded in 96-well plates and treated with Tunicamycin (TM) at different concentration. Then, the CCK-8 reagent (10 μL) was added in the medium and cells were incubated for another 4 h. Finally, the absorbance was measured at 450 nm.

### Transfection

The miR-627 or CALR overexpressing plasmids (miR-637 or CALR mimics and miR-637 inhibitors) and negative control were all synthesized from GenePharma (Shanghai, China). All plasmids were transfected into AGS cells using Lipo-2000 transfection reagent (Invitrogen, Carlsbad, CA, USA) according to the manufacturer's instructions.

### Dual-luciferase reporter assay

Wild-type CALR plasmid (CALR-WT) and mutant CALR plasmid (CALR-MUT) containing binding sites for miR-637 were integrated into the pGL3 promoter vector (GenePharma, Shanghai, China), and then transfected to AGS cells. The dual-luciferase reporter assay kit (Promega, Madison, WI, USA) was used to measure the luciferase activity.

### RT–PCR

Total RNA was extracted by Trizol reagent (Sigma-Aldrich, St. Louis, MO, USA). 1 μg RNA were used to synthesize cDNA and amplified by Quantitative real-time PCR using SYBR Green PCR reagent kits (Promega Corporation, Madison, WI, USA) according to the manufactures’ protocol. All samples were normalized to GADPH.

### Western blot

Total proteins of treated cells were extracted with RIPA buffer (supplement with protease and phosphatase inhibitor) and quantified with BCA protein assay kit (ThermoFisher Scientific, Waltham, MA, USA). Samples were separated by 10% SDS-PAGE and transferred onto PVDF membranes. The PVDF membranes were incubated with the primary antibodies and the level of each protein were visualized using an ECL system and normalized to β-actin. Primary antibodies were as follows: β-Actin (CST #8457, 1:2000), caspases 3 (CST #9662, 1:1000), cleaved caspases3 (CST #9664, 1:1000), caspases 8 (CST #9746, 1:1000), cleaved caspases 8 (CST #9748, 1:1000), CALR (CST #12238, 1:1000), CHOP (CST #2895, 1:1000), GRP78 (sigma #G9043, 1:1500).

### Statistical analysis

All data was shown as mean ± SD, and analyzed with SPSS software (Chicago, IL, USA). Statistical significance was evaluated by one-way ANOVA. *p* < 0.05 was defined as statistically significant.

## Results

### The level of miR-637 was decreased after TM treatment

**Figure 1.**
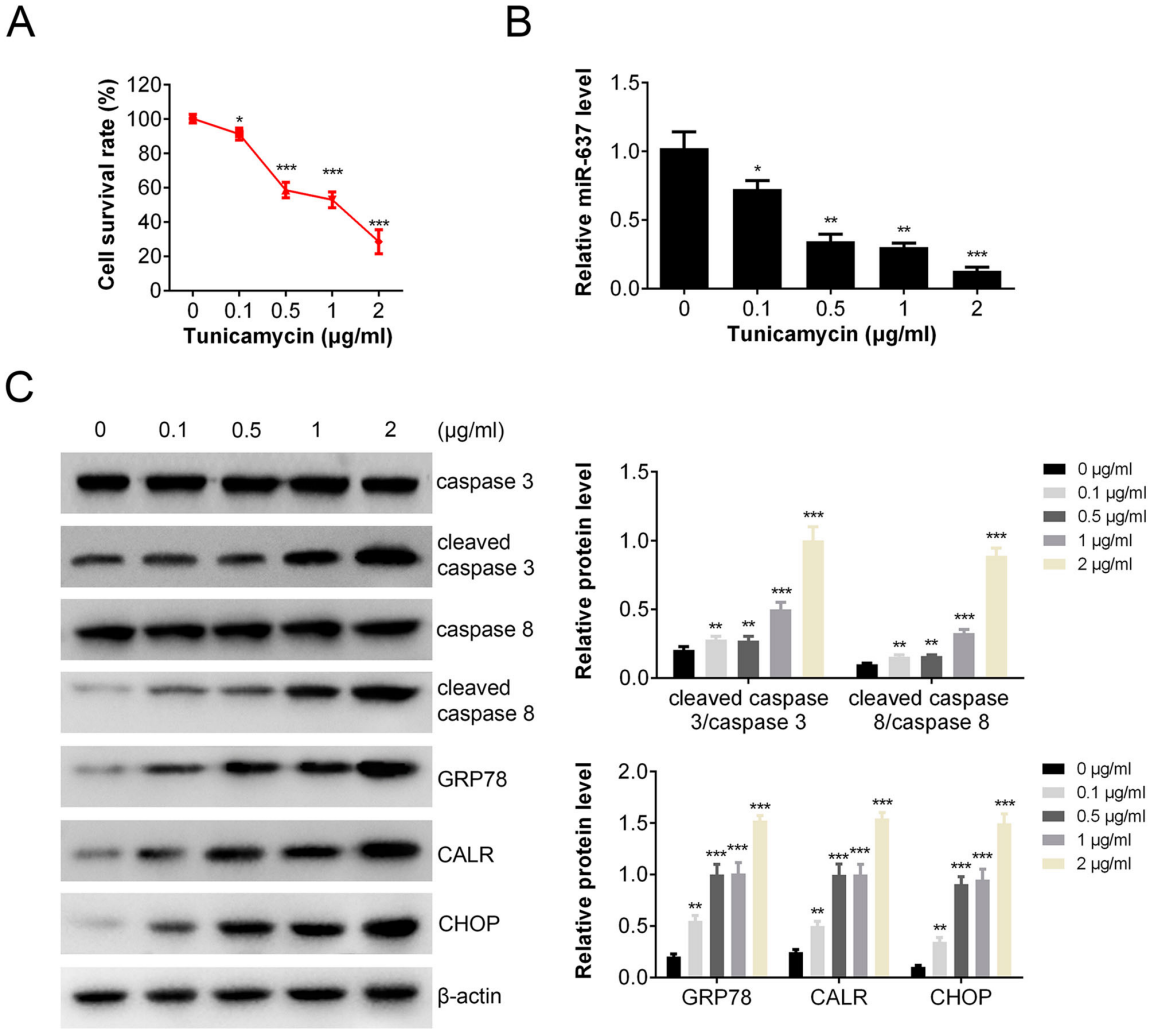
MiR-637 was decreased after TM treatment. AGS cells were treated with/without TM. Cell viability was analyzed by CCK-8 (A); level of miR-637 was analyzed by RT-PCR (B); cell apoptosis and ERS were tested by western blot analysis (C). **p < 0.01 compared with the control

To investigate the effect of ER stress on apoptosis, the viability of AGS cells (Human gastric cancer cells) was measured by CCK-8 assay after TM (ER stress inducer) treatment (with final concentration from 0 to 2 μg/mL). Results showed that cell survival rate decreased markedly from 100% to about 30% after TM treatment (Figure 1(A)). The transcription of miR-637 was also inhibited by TM in a dose-dependent manner (Figure 1(B)). To investigate the apoptosis and endoplasmic reticulum stress (ERS), the apoptosis and ERS associated proteins were measured by Western blot (Figure 1(C)). The levels of cleaved caspase 3/caspase 3 (caspase 3 activation) and cleaved caspase 8/caspase 8 (caspase 8 activation) were increased markedly in a dose-dependent manner after TM treatment, which indicated the pro-apoptosis effects of TM. The expressions of ERS related proteins (GRP78, CALR, CHOP) were all increased after treated with TM, indicating that ERS occurred.

### Overexpression of miR-637 promoted the apoptosis induced by TM

**Figure 2.**
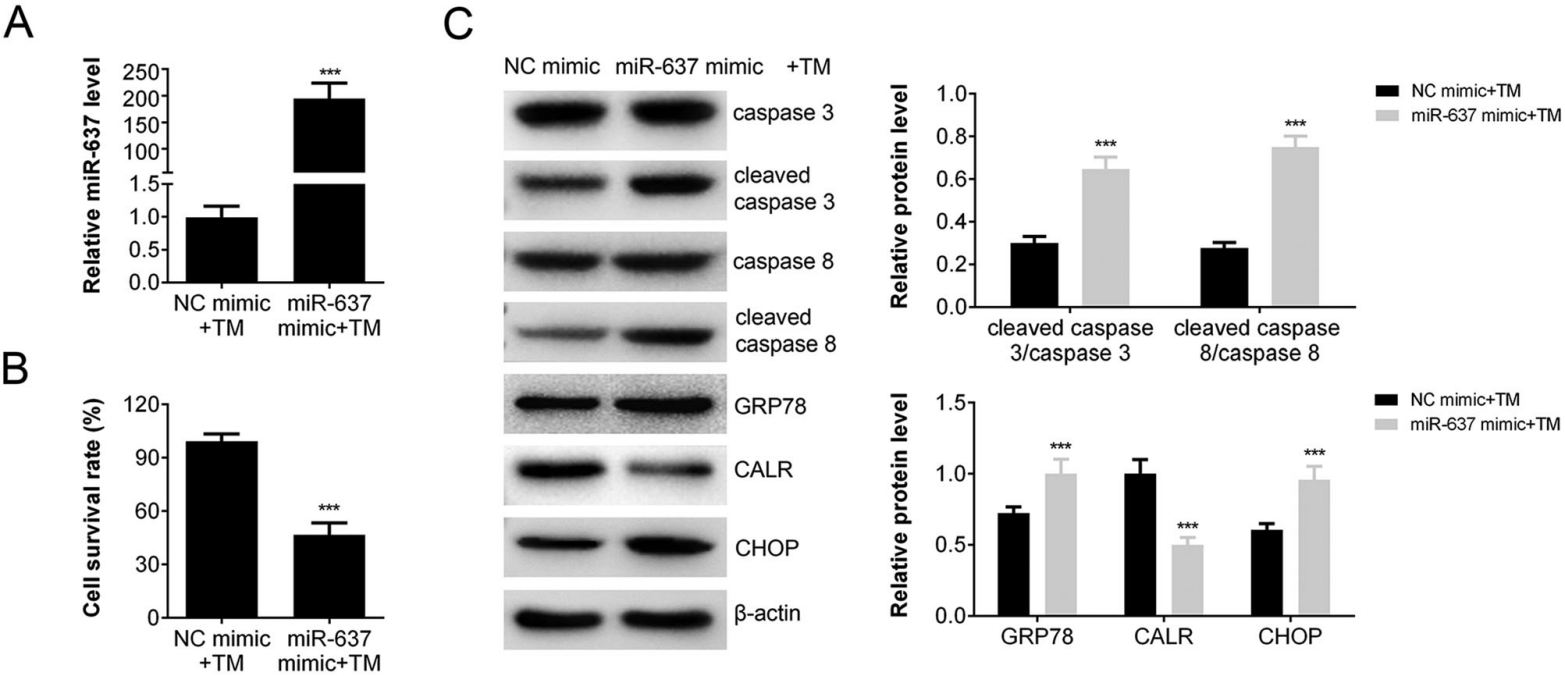
miR-637 Overexpression promoted the TM-induced apoptosis. (A) The level of miR-637 in AGS cells transfected with miR-637 mimics or negative control analyzed by RT-PCR; (B) The cell survival rate (transfected with miR-637 mimics or negative control) analyzed by CCK-8 assay. (C) The level of apoptosis and ERS associated proteins measured by western blot analysis.

To test the effect of miR-637 on apoptosis induced by TM, we constructed miR-637 over-expressing cells by transfection. The transfection efficacy was confirmed by RT–PCR (Figure 2(A)). The transcription of miR-637 in over-expressed AGS cells (miR-638 mimic + TM) was increased compared with that of negative control (*p *< 0.001 vs. NC mimic + TM). The amount of survival cells decreased about 50% of miR-637 over-expressing cells after treated with TM (*p *< 0.001) (Figure 2(B)), which indicated that miR-637 enhanced the apoptosis induced by TM. The level of cleaved caspase 3/caspase 3 and cleaved caspase 8/caspase 8 were increased in miR-637 over-expressing cells (Figure 2(C)), which was in accordance with the results of cell survival rate. The extent of ERS was also analyzed by Western blot. The expression levels of GRP78 and CHOP were increased in miR-637 over-expressing cells after treated with TM, while CALR expression was decreased (Figure 2(C)). These results indicated that miR-637 overexpression enhanced the TM-induced apoptosis and ER stress. In addition, it suggested that miR-637 may regulate the expression of CALR.

### MiR-637 regulated the expression of CALR

**Figure 3.**
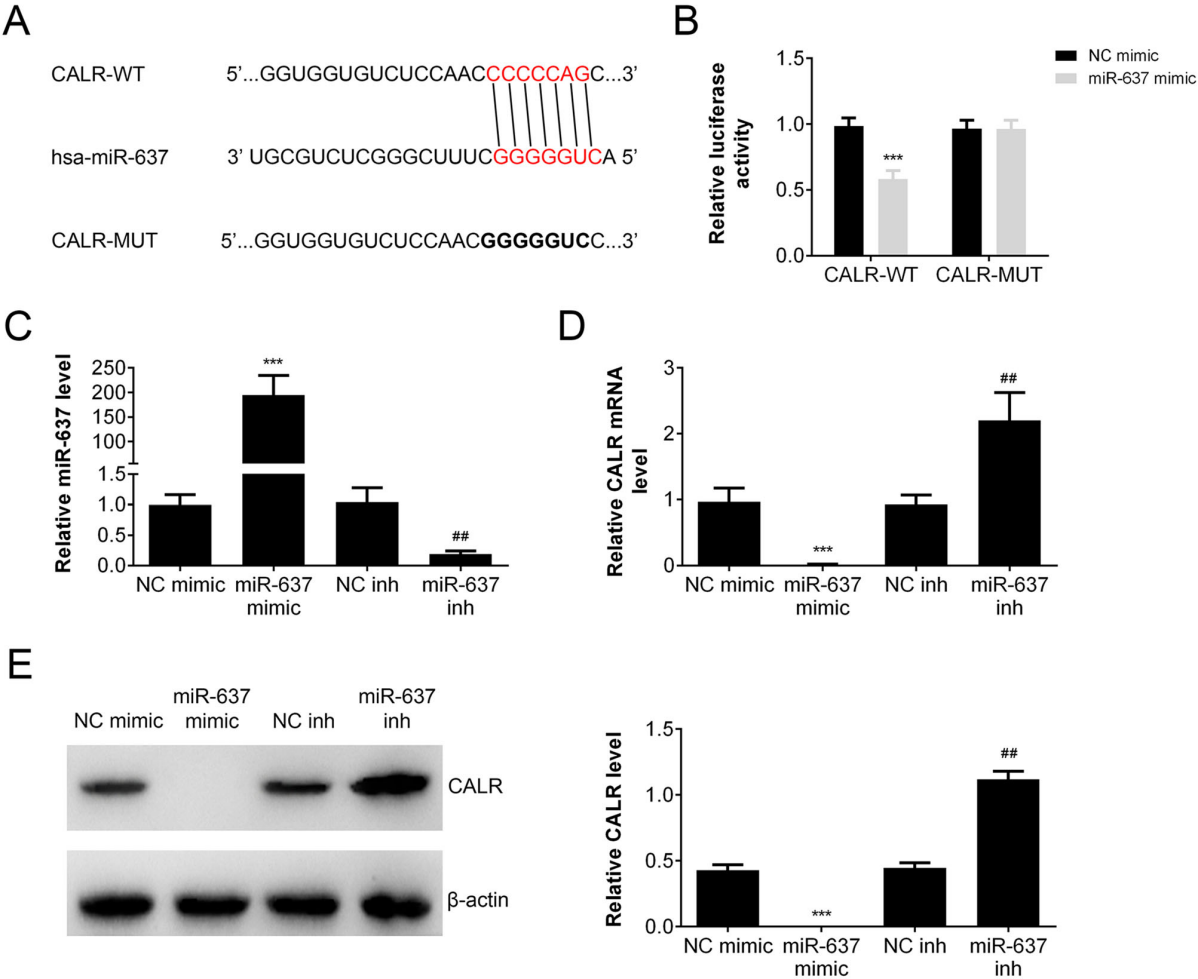
MiR-637 inhibited CALR expression. (A) Sequences of miR-637 and the potential binding site at the 3′-UTR of CALR. (B) The interaction between miR-637 and the CALR 3′-UTR tested by luciferase reporter assays. The level of miR-637 (C) and CALR (D) in miR-637 overexpression (miR-637 mimic) or repression (miR-637 inh) was analyzed by RT-PCR. The expression of miR-637 (E) and CALR (F) in miR-637 overexpression (miR-637 mimic) or repression (miR-637 inh) was analyzed by Western blot. ***p < 0.001 vs. NC mimic, ^##^*p* < 0.01 vs. NC inh.

Bioinformatic analysis (Targetscan: http://www.targetscan.org/) was employed to predict the target of miR-637. As shown in Figure 3(A), the sequence of miR-637 binds with the 3ʹ-UTR of CALR mRNA. To verify that CALR is one of the target genes of miR-637, a dual-luciferase reporter vector incorporating the 3ʹ-UTR of CALR was constructed and co-transfected into AGS cells with miR-637 mimic or negative control (miR-NC). The dual-luciferase activity assay (Figure 3(B)) showed that miR-637 showed inhibitory effect on the luciferase activity compared with NC mimic group in CALR wild type (CALT-WT) cells (*p* < 0.001), but not in CALR mutation (CALR-MUT) cells. To investigate the regulation of miR-637 on CALR expression, we constructed the miR-637 overexpressing or knockdown cells by transfection. The level of miR-637 was increased markedly in miR-637 mimic group (*p *< 0.001 vs NC mimic), while decreased in miR-637 inhibitor group (*p* < 0.01 vs NC inh) (Figure 3(C)). The transcription of CALR was inhibited by miR-637 overexpression (*p *< 0.001 vs NC mimic) and reversed by miR-637 inhibitor (*p* < 0.01 vs NC inh) (Figure 3(D)). The expression of CALR was similar with that of mRNA (Figure 3(E)), which indicated that miR-638 negatively regulated the expression of CALR.

### Overexpression of CALR in AGS cells reversed the pro-apoptosis effects of miR-637 overexpression in TM-treated cells

**Figure 4.**
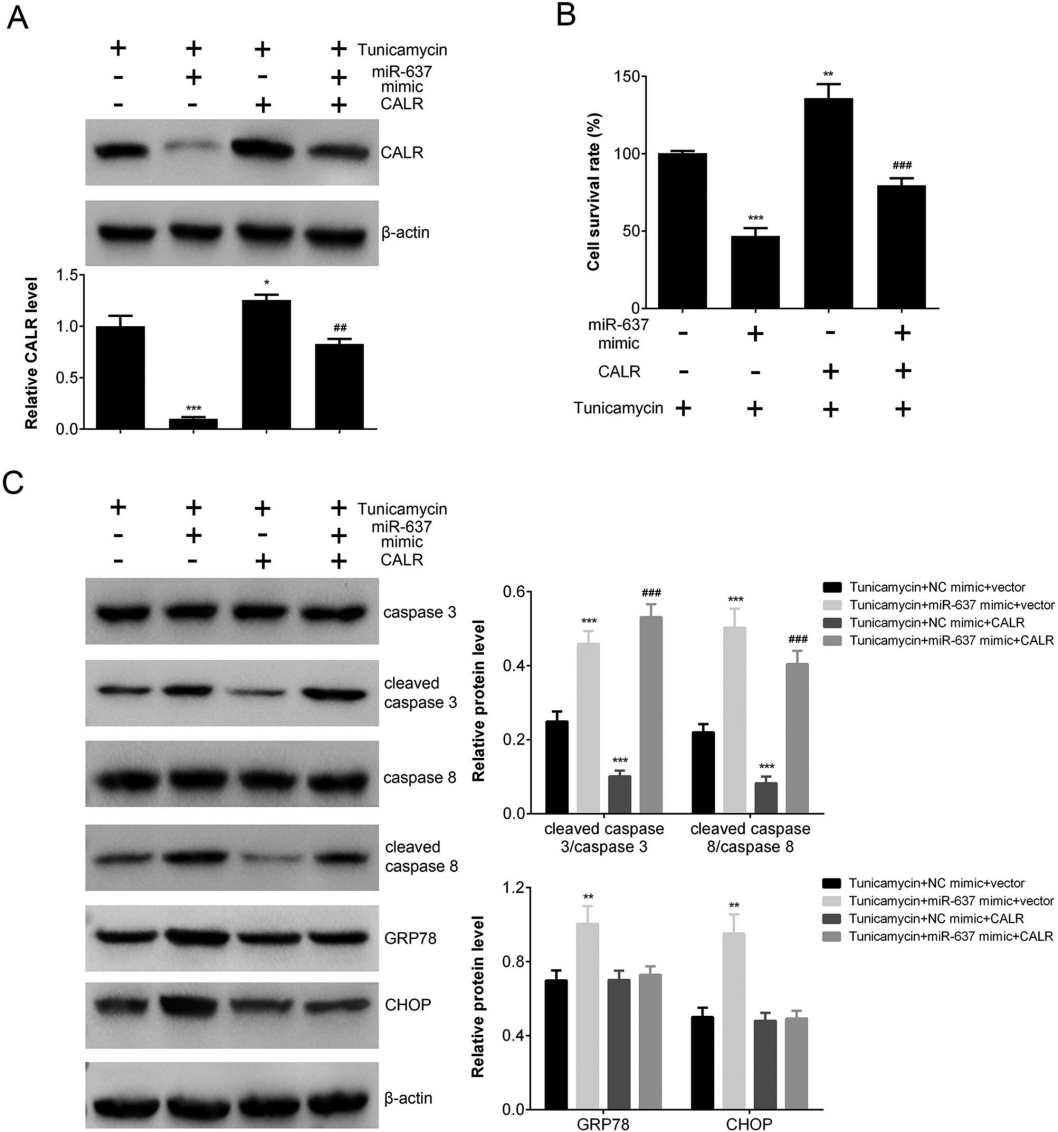
CALR overexpression reversed the pro-apoptosis effects of miR-637 in TM-treated cells. (A) The expression of CALR in AGS cells transfected with CALR and/or miR-637 mimic analyzed by Western blot. (B) The cell survival rate (when transfected with CALR and/or miR-637 mimics) analyzed by CCK-8 assay. (C) The level of apoptosis proteins analyzed by Western blot. **p < 0.01 and ***p < 0.001 vs. NC mimic, ^##^*p* < 0.01 and ^###^*p* < 0.001 vs. NC inh.

To investigate the effects of CALR on apoptosis and ERS induced by TM, and the regulatory effects of miR-637, we constructed the miR-637 or CALR overexpressing AGS cells, as well as co-transfected cells with both miR-637 and CALR. The results showed that expression of CALR was inhibited by miR-637, and CALR overexpression weakened the effects of miR-637 (Figure 4(A)). Cells of all group was treated by TM. The cell survival rate decreased in miR-637 overexpressing cells, while the effect was reversed by CALR overexpression in co-transfected cells (Figure 4(B)). The expression of ERS and apoptosis related proteins were analyzed by Western blot. The level of cleaved caspase 3/ caspase 3 was increased in miR-637 overexpressing cells, and decreased in CALR overexpressing ones. In co-transfected cells, CALR significantly reversed the effects of miR-637 on apoptosis (*p* < 0.001 vs. NC mimic+ CALR). The results of caspase 8 were similar with those of caspase 3, which indicated that CALR could reverse the pro-apoptosis effects of miR-637 in TM-treated cells. The expression of ERS associated proteins (GRP78 and CHOP) was increased in miR-637 overexpressing cells, but not in CALR overexpressing cell (Figure 4(C)). These results indicated that CALR could reverse the pro-apoptosis effects of miR-637, and decrease ER stress.

## Discussion

Gastric cancer, a leading cause of cancer mortality all over the world, has a higher incidence in Asia including China (Rugge et al. 2015). Recently, researchers reported that the aberrant levels of miRNAs may be hallmarks of gastric cancer (Chang et al. 2011; Li et al. 2012). Shin and Chu reviewed the gastric cancer-related miRNAs and the target genes in tumor progression, and indicated the various effects on cancer cells including proliferation, differentiation, apoptosis, and invasion (Shin and Chu 2014). MiRNAs could be a potential tool for cancer therapy. Previous studies reported that the expression of miR-637 was abnormal in ovarian cancer patients, and *in vitro* research confirmed that miR-637 could inhibit tumorigenesis for many kinds of cancer cells, including hepatocellular carcinoma, breast cancer and thyroid carcinoma (Yuan et al. 2018; Wang et al. 2019; Zhang et al. 2019). However, the role of miR-637 on gastric cancer is still unclear.

First, we found that the level of miR-637 in AGS cells decreased markedly after TM treatment, which indicated that miR-637 enhanced ER stress-induced apoptosis. In addition, the following study showed that miR-637 overexpression could significantly enhance TM-induced apoptosis of AGS cells. Other paper confirmed that miR-637 can markedly suppress cell proliferation or induce apoptosis, which was in accordance with the present study. For example, in pancreatic ductal adenocarcinoma, miR-637 over-expression significantly suppressed cell proliferation and induced apoptosis through inhibiting expression of Akt1 (Xu et al. 2018).

Meanwhile, as TM promotes apoptosis through endoplasmic reticulum stress (ERS) and the following DNA synthesis blocking, we hypothesized that miR-637 was related to apoptosis induced by ERS. CALR, a high capacity Ca2+ binding protein of ER and SR, plays a role in protein folding and homeostasis of Ca2+. The alteration of CALR may cause ER stress-induced apoptosis. Research found that CALR silencing prevented Epibrassinolide (EBR, a member of brassinostreoids plant hormones)-induced UPR and apoptosis in colon cancer cells, indicating that CALR inhibited EBR induced apoptosis through activating ER stress (Obakan-Yerlikaya et al. 2017). Our results also showed that expression level of CALR in AGS cells was increased after treating with TM. Moderate ER stress plays an important role in cellular homeostasis and cells survive. However, When ER stress destroys protective cellular responses, apoptosis ensues. CALR acts as an ER molecular chaperone and regulates a variety of cellular functions including apoptosis (Jo et al. 2017). Besides, through bioinformatics, we found that miR-637 could bind the 3ʹ-UTR of *CALR*, which indicated that miR-637 may regulate apoptosis partly through targeting *CALR*. CALR transfection reversed the effects of miR-637 overexpression, which further proved that miR-637 negatively regulated the expression of CALR, and the following apoptosis. That is to say, overexpression of miR-637 artificially disrupts the ER stress of cells, resulting in decreasing ability to cope with external stress and thus apoptosis. Many researches confirmed that CALR was related with cancer cell apoptosis, but miR-637 was not the only non-coding RNA to regulate CALR. For example, researchers found that miR-27a could affect drug-induced apoptosis of human colorectal cancer cells through targeting CALR (Colangelo et al. 2016). In addition, miR-637 could also affect cancer cells through other ways. Zhang et al. reported that miR-637 regulated the proliferation and metastasis of human keloid fibroblast cells (HKF) by Smad3 signaling pathway, and miR-637 downregulation aggravated the illness (Zhang et al. 2018). To sum up, miR-637 could inhibit apoptosis of cancer cells through negatively regulating the expression of CALR.

In conclusion, the present study suggests that miR-637 participates in the ER stress-induced apoptosis in gastric cancer cells by suppressing CALR expression. miR-637 or CALR may be a future potential target for gastric cancer treatment.

## Data Availability

All data generated or analyzed during this study are included in this published article.
